# Safety and Efficacy of a New Smartphone-controlled Vibrating Capsule on Defecation in Beagles

**DOI:** 10.1038/s41598-017-02844-4

**Published:** 2017-06-06

**Authors:** Jin Yu, Yang-Yang Qian, Chao-Hui He, Shu-Guang Zhu, An-Jing Zhao, Qian-Qian Zhu, Cheng-Wei Shao, Tie-Gong Wang, Yang Wang, Gui-Ling Ding, Zhuan Liao, Zhao-Shen Li

**Affiliations:** 1 Department of Gastroenterology, Changhai Hospital, Second Military Medical University, Shanghai, China; 2 Department of Gastroenterology, the Fifth Affiliated Hospital of Zunyi Medical College, Changhai Hospital, Second Military Medical University, Shanghai, China; 3 Department of Radiology, Changhai Hospital, Second Military Medical University, Shanghai, China; 4 Department of Pathology, Changhai Hospital, Second Military Medical University, Shanghai, China

## Abstract

Constipation, mainly manifesting as abdominal discomfort and painful defecation, is considered as a chronic disorder. Due to a lack of effective therapy, it imposes a significant economic burden and greatly impacts patients’ quality of life which prompt searches for new, original approaches. Based on the research of vibrating capsule (VC) carried out by Ron *et al*., we investigated the safety and efficacy of an innovative, multi-mode VC in terms of its effect on defecation in animal studies. The parameters associated with different operation modes of VCs can be detected and adjusted by smartphone controlled external configuration device (ECD). The results of blood tests, physiological parameters, CT scan and pathological examination showed no significant abnormality, which undoubtedly confirmed the safety of VCs. For efficacy studies, defecation frequency of beagles increased after administration of these capsules without influence on stool characters. Meanwhile, the mean time of capsule evacuation tended to be reduced while showing no significant difference between different modes. In summary, this study elucidates the safety and effectiveness of VC in prompting the passage of gastrointestinal walls thus greatly increasing the defecation frequency. This study innovatively displays the promising application of VC in the treatment of constipation.

## Introduction

Constipation, a common cause of abdominal discomfort and painful defecation, is defined as the less-frequent passage of stool or the passage of hard stool^1^. Since most of the patients with constipation have symptoms for many years, it is considered a chronic disorder^2^. Even though a minority of those with constipation seek medical treatment, it still accounts for 8 million annual visits to physicians in the United States^3^. In a systematic review on the global prevalence of idiopathic constipation, Suares and Ford reported a pooled prevalence of 14%^4^. It is revealed that the prevalence over 60 can be up to 22%, and is estimated to be as high as 30% in selected populations especially in women, non-whites, those aged >65 years and those with lower socioeconomic status^5^. With high prevalence, it imposes a significant economic burden in direct healthcare costs as well as having a significant impact on patients’ quality of life and working productivity^6, 7^.

Based on current schemata, chronic constipation is usually classified into 2 categories: idiopathic (slow-transit and normal-transit) and secondary constipation^8^. There are many evidence-based therapeutic approaches to the treatment of chronic constipation. World Gastroenterology Organization(WGO) advocates a graded approach to treatment from changes in lifestyle and diet, which includes osmotic laxatives to stimulant laxatives, enemas, and prokinetic drugs. Similarly, the American Society of Colon and Rectal Surgeons regards fiber, fluid supplementation and osmotic laxatives as initial management, and stimulant laxatives as second-line treatment. Surgical management is considered for refractory colonic slow-transit constipation^1, 5, 9, 10^. While there are many therapeutic options available, the combination of high prevalence, side effects of treatment, high recurrence rate and low patient satisfaction level has prompted searches for new, original approaches.

In 2015, Ron *et al*.^11^ carried out research to validate the safety and potential efficacy of a vibrating capsule in the treatment of constipation. The capsule was activated using an electromagnetic signal and the fixed vibrating sequence (A or B, as recommended by the physician) began after a predetermined delay of 6–8 h to allow the capsule to arrive at the colon. It reported no serious adverse events and minor adverse effects were transient. A significant increase in the mean number of bowel movements was seen in 23 of the 26 patients.

A new kind of vibrating capsule with four vibration modes has been developed. This new vibrating capsule may be modulated by a smart-phone controlled external configurator. Compared with that in previous studies, our capsule is directed and activity-controlled externally.

This study investigated the safety and efficacy of an innovative, multi-mode vibrating capsule in terms of its effect on defecation in animals. The main focus was to determine whether the administration of smart phone-controlled VC was feasible in the treatment of constipation with its parameters being detected and adjusted by smartphone externally.

## Results

### Safety study

#### Results of physiological and blood parameters

In the first animal study, eight dogs each swallowed three vibrating capsules. In all cases, the dogs’ behaviour and mental status were normal with no signs of nausea, vomiting or oral expulsion of capsules during the follow-up period. After the vibrating sequence began, vibration of the abdomen can be perceived and varied with modes. It was feasible to detect and define the capsule’s activity parameters by the external smartphone-controlled configurator. Of the 24 administrated capsules, all were evacuated two days after ingestion. There was no problem with expulsion of the capsules and capsules were expelled with no morphological abnormality.

After administration of different capsules, there were no clinically significant abnormalities in any of the psychological and blood parameters(P > 0.05).

#### Pathological examination results

**Figure 1:**
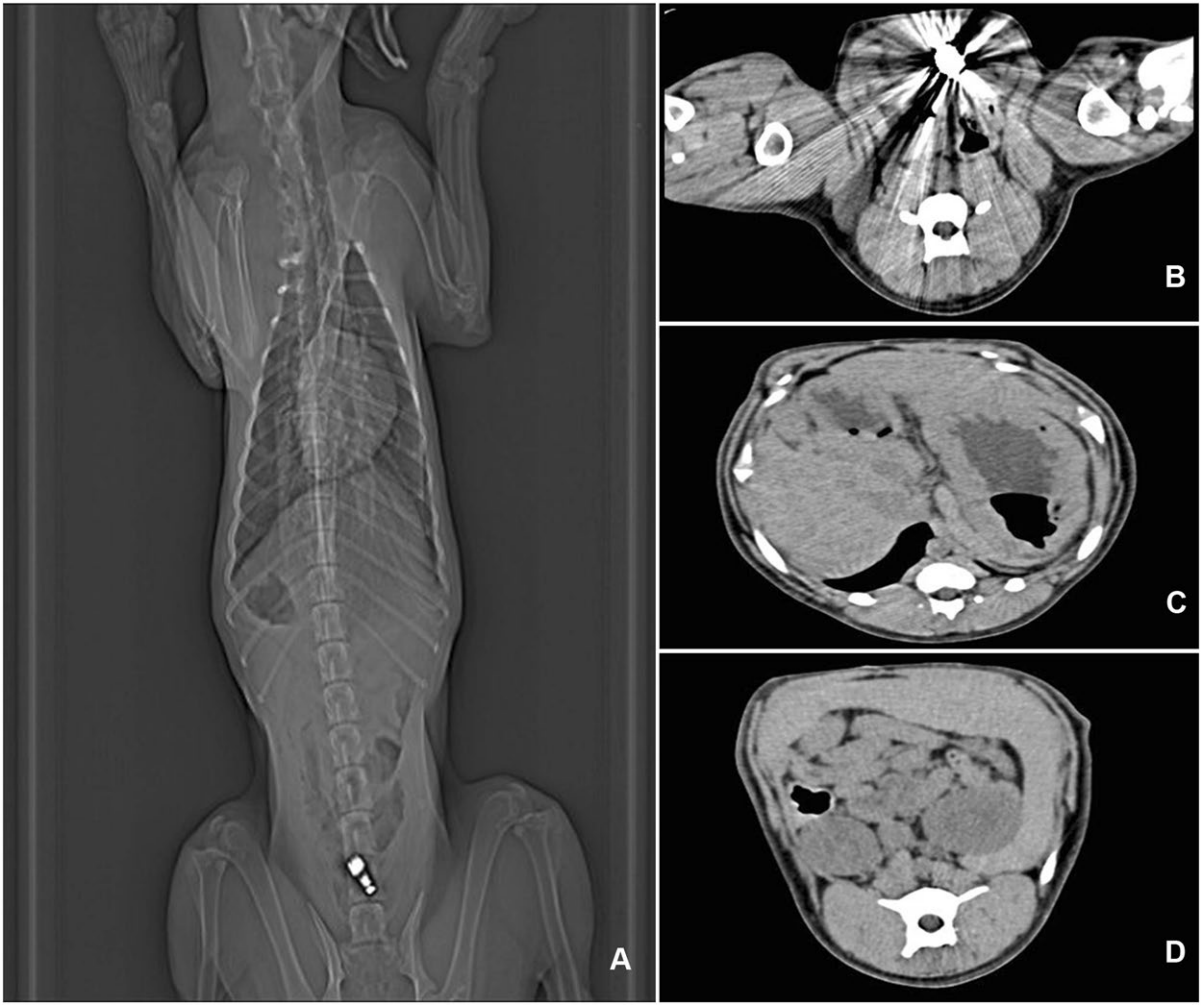
Radiographic examinations after administration of ultra-high frequency capsules. (**A**) Abdominal plain film after 2 hours’ vibration at ultra-high frequency. (**B**) CT image of stomach (arrow showing VC in the stomach). (**C**) CT image of intestine. (**D**) CT image of rectum.

**Figure 2:**
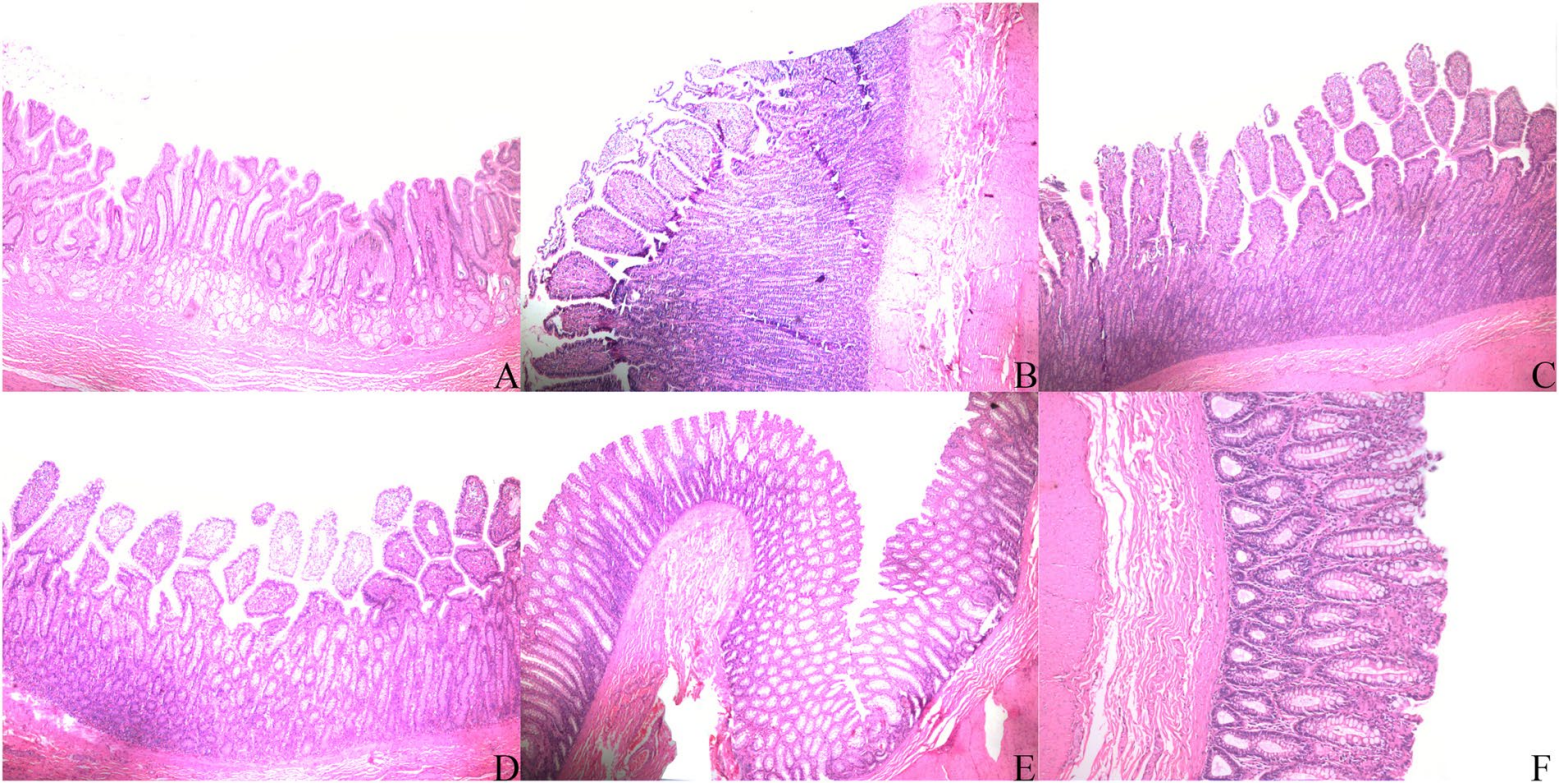
Pathological sections of integrated gastrointestinal wall after capsule administration. None reported lesion or abnormality. (**A**) Stomach. (**B**) Duodenum. (**C**) Jejunum. (**D**) Ileum. (**E**,**F**) Colon.

After two hours’ vibration at ultra-high frequency, no signs of intestinal obstruction and perforation were seen on CT scan (Fig. 1A–D). There was no evidence of perforation, ulceration or bleeding macroscopically in the pathological specimens. Parasites were noticed in the small intestine of one dog. There was no microscopic evidence of interstitial edema, glandular distortion, obvious inflammatory cell infiltration and dysplastic epithelium in any of the pathological specimen (Fig. 2A–F).

### Efficacy study

No erythrocytes, leukocytes, macrophages, residual chyme or blood was observed in routine stool examination. Meanwhile, fecal transferrin and occult blood test were both negative.

#### Defecation frequency

**Figure 3:**
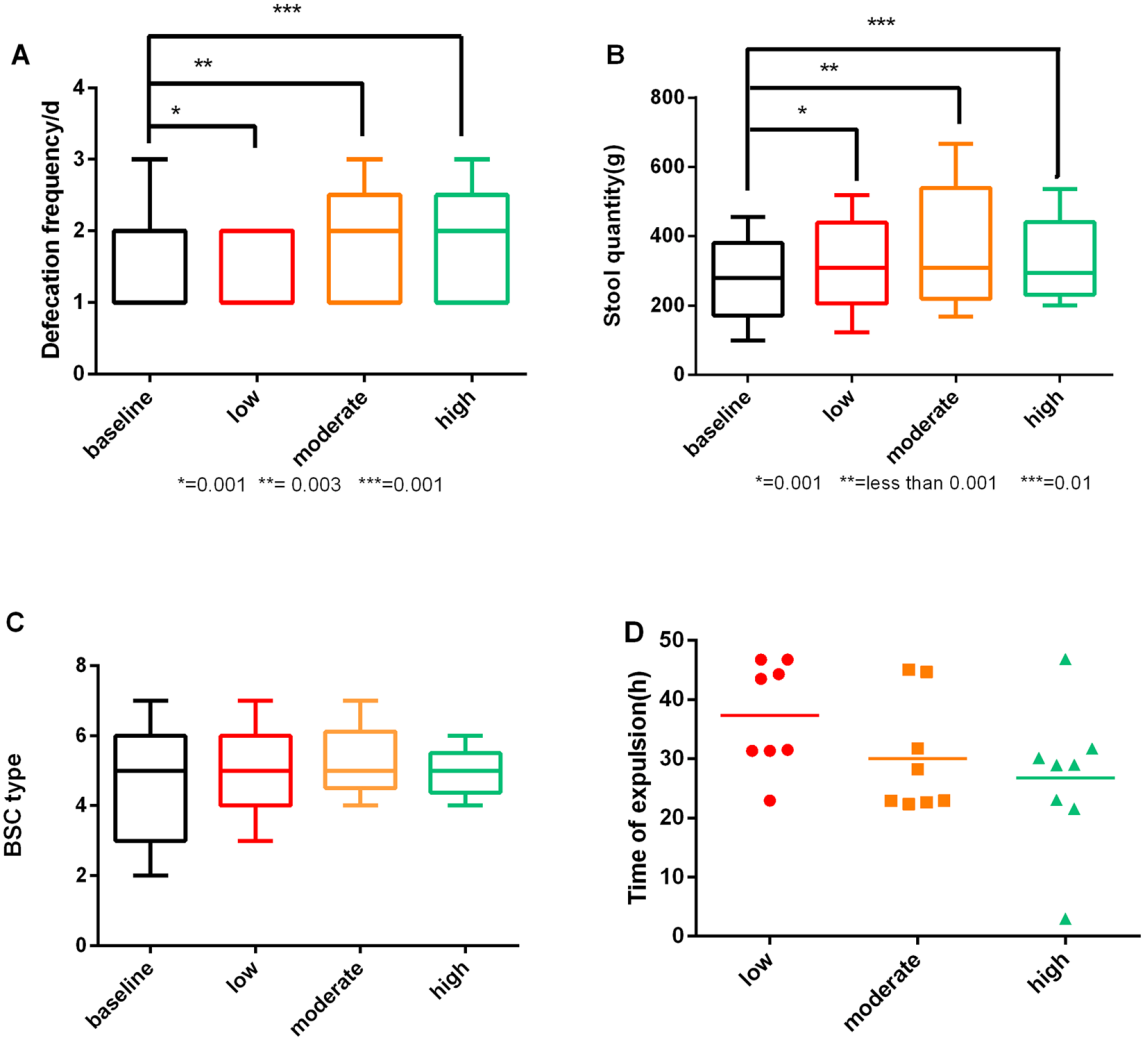
Efficacy study results. (**A**) The median defecation frequency increasing greatly after administration of VCs at different modes compared with that at the baseline. (**B**) VC administration proved to improve stool quantity. Median values were denoted by the central horizontal line within the boxplots containing IQR for the results. (**C**) VC administration did not change the faecal character which showed its safety. (**D**) Mean expulsion time of capsules at different modes depicted by the horizental line showing no significant difference. (dots, square, triangle indicating beagles swallowing low, moderate and high mode VCs, respectively).

In the main efficacy endpoint, there was a significant increase in the frequency of defecation (/day) after capsule ingestion. The median frequency at baseline, low, moderate and high mode were 1 (IQR 0), 2 (IQR 1), 2 (IQR 1) and 2 (IQR 1), respectively (Fig. 3A). The median frequency of defecation was higher than that at the baseline after administration of low (P = 0.001), moderate (P = 0.003) and high mode vibrating capsules(P = 0.001), while there was no statistical difference among these three modes (all P > 0.05).

#### Stool quantity

Vibrating capsules of different frequency all can increase stool quantity. The median stool quantity at baseline, low, moderate and high mode were 280.55 g (IQR 62.63), 308.6 g (IQR 70.88), 309.3 g (IQR 140.8) and 294.8 g (IQR 81.62), respectively (Fig. 3B). Significant difference was shown between dogs in each VC frequency group (P < 0.001). Stool quantity increased greatly from baseline after administration of vibrating capsules (P = 0.001, <0.001 and 0.01 for low, moderate and high frequencies). Again, no statistical difference existed between each pair of the three modes (all P > 0.05).

#### Faecal character

The Bristol Stool Chart (BSC) allows patients to identify their stool form using different images with accompanying written descriptors^12^. According to Bristol Stool Chart, stool form can be classified into 7 types. The median scores of BSC for baseline, low, moderate and high frequency VCs were 5(IQR 1), 5(IQR 0), 5(IQR 0) and 5(IQR 0) (Fig. 3C). No significant difference was shown in stool character between dogs. Similarly, compared with that at baseline, stool character at low, moderate and high frequency existed no prominent abnormalities (all P > 0.05). Meanwhile, no significant changes of stool character were observed between each pair of the three modes.

#### Time of expulsion

The mean time of expulsion were 37.32 h, 30.07 h and 26.77 h for low, moderate and high frequency VCs, presenting a descending trend (Fig. 3D). In the safety study for ultra-high frequency mode, VCs were found in rectums of six dogs while the other two remained in stomach, with parasites showing up in the small intestine of one dog and the other one stopping working due to mechanical troubles. The mean time for eight dogs to expel the capsules showed no statistically difference (P = 0.346), and neither did the expulsion of capsules at different modes (P = 0.137).

## Discussion

Chronic constipation is a disabled disorder affecting approximately 20% of the world’s population. Although considered benign in most cases, chronic constipation can result in chronic illness which can greatly influence the quality of life including fecal impaction, incontinence, hemorrhoids, anal fissures, bleeding, and in the most extreme cases, colon perforation^13^. Yet the pathogenesis of constipation is still unclear. Given the present situation and advances in treatment, a new innovative vibrating capsule has been developed. With the advantage of easy operation, individualization, low cost and no obvious side effects, they can impinge on the gastrointestinal walls physically, inducing increased peristaltic activity that propel the bowel content through bowels, thus relieving constipation.

In the safety studies, through analyses with the mental status, physiological, blood, stool parameters, CT scan as well as pathological anatomy of dogs, vibrating capsules proved to be highly safe, which is consistent with the study carried by Ron *et al*. With the assistance of smartphone and wireless configurator, the remaining power can be measured and the frequency of vibrating capsules can also be precisely adjusted as needed externally.

In terms of efficacy studies, the results of the experiment showed a statistically significant increase in the defecation frequency and stool quantity after dogs swallowed VCs, while no significant difference existed in stool character, which confirmed the safety and effectiveness. But there was no significantly difference of defecation frequency and stool quantity between multiple modes, which might have something to do with heterogeneity of dogs.

There was a trend towards a reduction in VC expulsion time with increasing vibration frequency. In the safety study of ultra-high frequency mode, VCs were found in rectums of six dogs after vibrating for three to four hours, which greatly shortened the expulsion time compared with high frequency mode. Given the small sample size, no statistically difference was observed. Thus further studies and constipation models are needed to confirm these issues.

In conclusion, VCs proved to be safe and effective in animal studies and it is feasible for smartphone to control VCs externally. By mechanical stimulation of the gastrointestinal walls, capsules can greatly promote defecation frequency without any abnormality in stool character. Meanwhile, the mean time of expulsion had a descending trend with frequency of VCs increased. Ron *et al*. has depicted that vibrating capsules can greatly improve the mean number of bowel movements^11^. Given the defecation promotion effect of VC illustrated by our study, we assumed that VCs may greatly relieve constipation. Thus we will further our research to validate the clinical application of VCs in constipation management with these encouraging results.

## Methods

### Study object

Experimental subjects are all quarantine qualified beagles, purchased from Shanghai Jiagan Biological Technology Co. Ltd (female: male = 1: 1), with 30–40 cm in height and 9.3 ± 1.7 Kg in weight (laboratory animal production certificate No.SCXK 2010-0028).

Dogs were acclimatized for 10–15 days to exclude stress involvement. During the acclimatization period, eight dogs were raised in stainless cages respectively at 19–26 ± 4 °C with humidity 40–70%, gas change 8–10/h and day and night ratio 12 h/12 h. Each dog had free access to 450 g food and 1500 ml water daily.

### Study site

The study was conducted in the animal laboratory center of Second Military Medical University (animal laboratory licence No. SYXK 2012-0003).The experimental protocol was approved by Laboratory Animal Ethics Committee of Second Military Medical University (animal ethical review No.13071002121). All experiments were conducted in accordance with the National Institute of Health Guide for the Care and Use of Laboratory Animals.

### Study intervention

The intervention apparatus is composed of the capsule itself and an external configuration device (ECD). The capsule is developed by ANKON Medical Technology Co. Ltd., with 26.7 mm in length, 11.8 mm in diameter and 4.5 ± 0.5 g in weight. The vibration of the capsule can be set at four different modes by pushing the corresponding buttons on the ECD, that is dormancy, low, moderate and high. The capsule stops vibrating at the dormancy mode, and the vibration frequency ratio among the mode of high, moderate and low is 1:0.8:0.5°.

**Figure 4:**
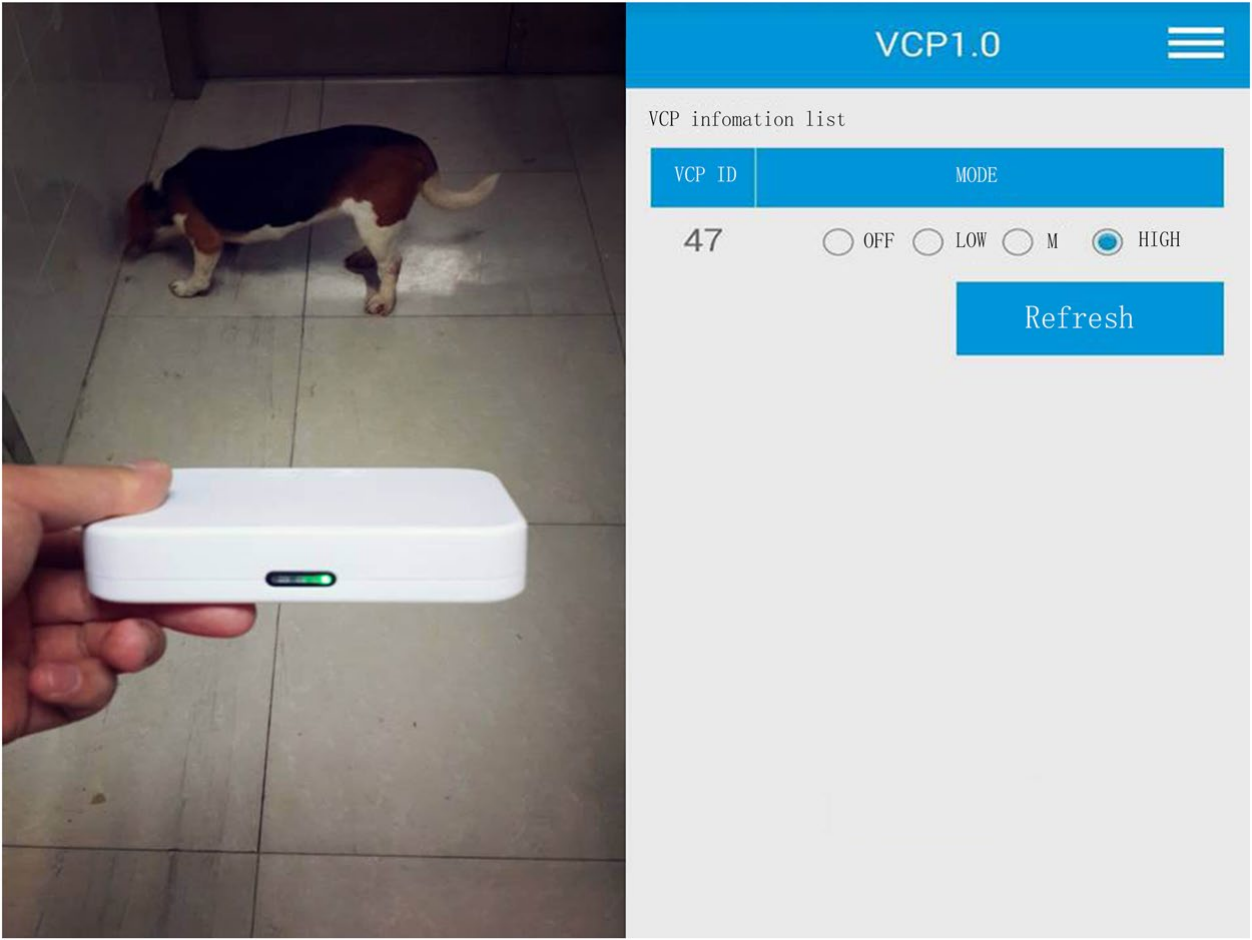
Activity of capsules can be detected and adjusted by configurator and configurator-matched App externally.

**Figure 5:**
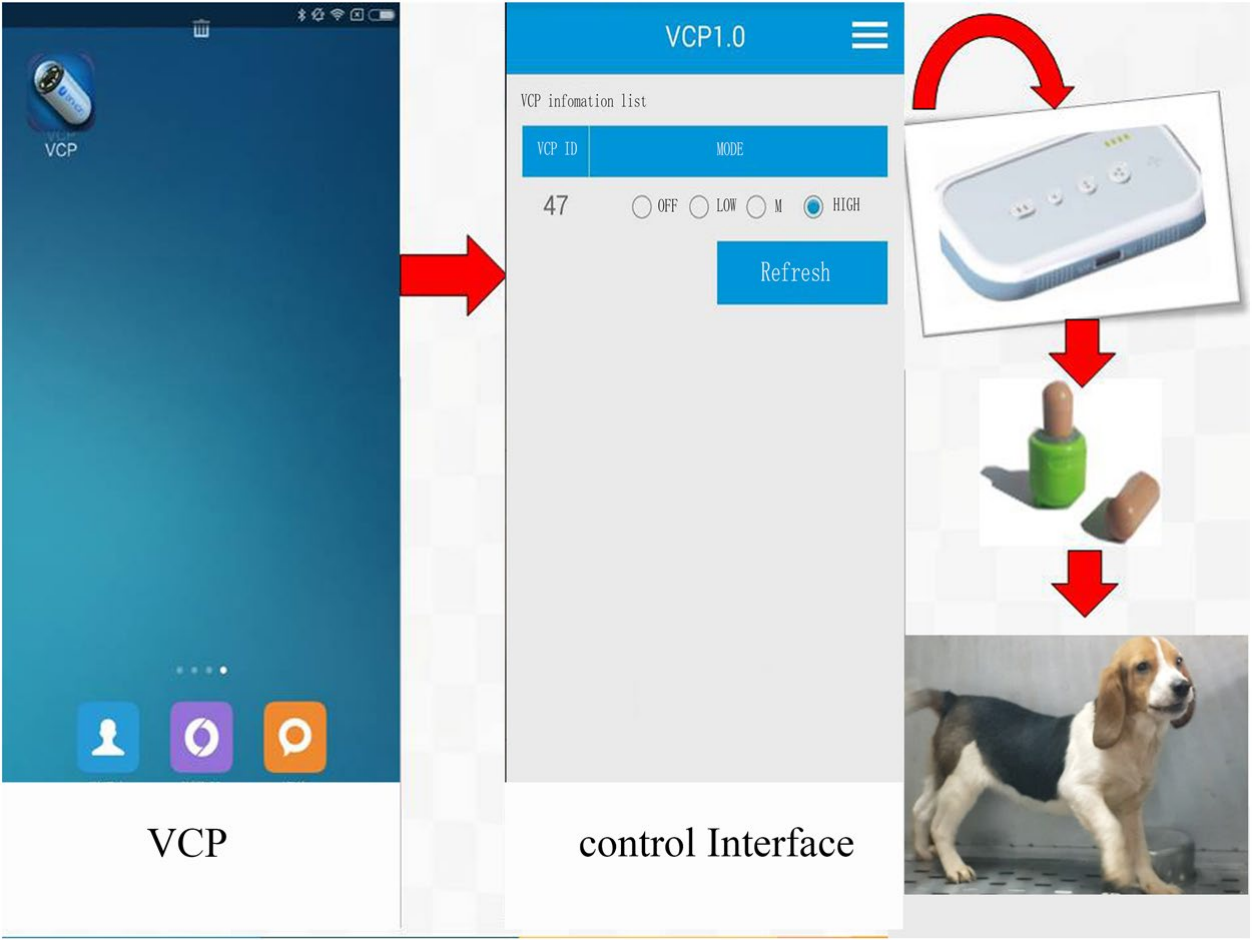
Diagram of vibrating capsule and external configuration device.

There is a two-way RF communication between VC and ECD, thus allowing VC to generate abrupt vibration by an eccentric motor. Once the ECD is put near the abdomen, the vibration mode can be activated and changed by the ECD. Moreover, a smartphone can communicate the ECD through an APP called VCP, which makes it feasible to select the mode and define the activity parameters of VCs externally. Semiconductor chip inside each VC with unique serial number is for recognition and manipulation (Figs 4 and 5).

### Study measurements

#### Physiological parameters

Continuously recorded physiological parameters including temperature (°C), heart rate (bpm), respiration (times/m), weight (Kg), conscious status and behaviour (normality, somnolence, stupor, coma, nervousness, restlessness, laziness, barking, groaning, aggression, etc.), daily intake (g) and daily drink (ml) of beagles for 5 days.

#### Blood parameters

Fasting venous blood was examined for white blood cell count (WBC), red blood cell count (RBC), hemoglobin (Hb), platelet count (PLT),alanine transaminase (ALT), aspartate aminotransferase (AST), total bilirubin (TBIL), direct bilirubin (DBIL), total protein (TP), albumin (ALB), urea nitrogen (BUN), serum creatinine (SCR), potassium (K) and sodium (Na).

#### Stool parameters

Stool parameters recorded were consisted of daily defecation quantity(g), defecation frequency and stool characters. Morning fecal specimen were taken for routine and occult blood test.

### Study Design

#### Study at normal mode

In the first animal study, eight dogs began to fast at 20:00 pm the day before the program, and all the psychological and stool parameters were recorded 12 hours later. At 9:00 am, each of the eight dogs initially swallowed one capsule. Then dogs had free access to food and water. The vibrating sequence began at low frequency after a predetermined delay of 2 h to allow the capsule to pass through the pylorus. Researchers observed and reported all the parameters needed at 11:00, 14:00, 17:00 that day and at 08:00, 14:00, 17:00 every day thereafter until the expulsion of the capsule was confirmed in the stool collection kit. Appearance of capsules, stool characters, quantity and color all needed close observation and record. The day after administration of capsules, blood routine, biochemical panel, stool routine and occult blood test were reviewed. After the expulsion of first capsule was confirmed, another one would be given at least 3 days later for the investigation of other modes.

#### Safety study at ultra-high frequency mode

The vibrating sequence was set at ultra-high frequency and the previous steps were repeated for capsule administration. Abdominal CT scan was performed after two hours’ vibration. Thereafter, with dogs euthanized and dissected, gastrointestinal explorations were carried out immediately. Biopsies with specimen of 3 cm x3 cm in size were obtained wherever any possible macroscopic lesions were identified.

### Statistical analyses

Data were presented as median with interquartile range (IQR) for continuous variables. Statistical analyses were conducted using SAS” version 21.0. The covariance analysis was applied for testing the statistical significance of changes between the baseline assessment and the multiple parameters in treatment period with a final two-sided P value of less than 0.05 indicating statistical difference.
